# Do Whale Sharks Select for Specific Environments to Give Birth?

**DOI:** 10.1002/ece3.70930

**Published:** 2025-02-16

**Authors:** Freya C. Womersley, Matt J. Waller, David W. Sims

**Affiliations:** ^1^ Marine Biological Association The Laboratory Plymouth UK; ^2^ Ocean and Earth Science, National Oceanography Centre Southampton University of Southampton Southampton UK

## Abstract

Neonate whale sharks < 1.5 m in length are rarely encountered, with approximately 35 sightings recorded globally between 1970 and 2020. Although potentially pregnant females seem to frequent certain sites, parturition areas are unknown, and most neonates have been sighted opportunistically in offshore environments, suggesting nursery habitat may occur in remote parts of the ocean. Here, documented accounts of neonate whale sharks with corresponding locations were mapped in relation to oceanography to identify whether there are commonalities in where they occur. Results show that locations of neonate sightings coincide with permanent oxygen minimum zones (OMZs)—with associated high surface chlorophyll‐a (Chl‐a) and low oxygen at depth—more often than would be expected by random chance. Two main hypotheses are proposed to explain this apparent association: (i) adult female whale sharks selectively pup in waters adjacent to low oxygen regions offering a proximate refuge from oceanic predators as well as enhanced foraging opportunities, or that (ii) pupping occurs randomly in the open ocean but that OMZs restrict neonates to shallower surface waters where they are more frequently encountered by humans than elsewhere. Testing both hypotheses requires more data on the relationship between whale shark movement ecology and dissolved oxygen concentrations. As a first step, a model predicts the highest likelihood of neonates occurring in waters above OMZs, focussed around intermediate Chl‐a regions at the boundaries of highly productive upwelling systems. These areas could be the focus of future, more targeted studies. Here, biologging devices measuring in situ oxygen concentrations will be useful for exploring how different life stages interact with OMZs, which are expanding due to climate‐driven deoxygenation. What this might mean for neonate whale shark conservation in future warmer oceans remains an open question.

## Graphical abstract

Little is known about the broad ecological value of extreme environments such as low‐oxygen waters for mobile animals like pelagic sharks. Here we find that whale shark—the world's largest fish—neonate sightings coincide with higher surface chlorophyll‐a and lower oxygen at depth more often than would be expected by random chance. Two main hypotheses are proposed to explain this apparent association, and avenues for future research are suggested to close data gaps.

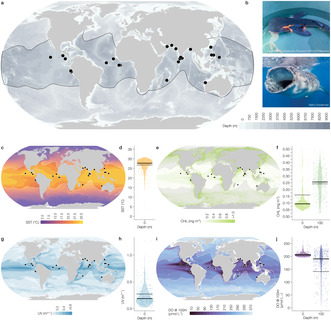

## 1 Introduction

The global ocean is warming, acidifying and deoxygenating due to anthropogenically driven climate change (Gruber et al. 2021). Marine megafauna may be more resilient to these environmental shifts due to their high‐mobility, wide‐ranging behaviour and often broad thermal niche. Pelagic sharks, for example, are known to utilise extreme environments for foraging opportunities, including use of extremely low oxygen waters (Vedor et al. 2021; Waller et al. 2024). However, these areas can also be exploited by humans (Vedor et al. 2021), are increasingly subject to stressors acting in conjunction with one another (compound events) (Li et al. 2024) and are projected to undergo further, chronic changes in future (Breitburg et al. 2018). How animals use such habitats across life history stages therefore governs the population scale impacts of climate change and exposure to human sources of stress that can potentially result in direct mortalities (Waller et al. 2024). Of particular importance is the role of these habitats in reproduction. For instance, whether pelagic sharks use extreme environments to give birth and, in doing so, potentially maximise foraging opportunities whilst also reducing predation remains poorly understood.

Whale sharks, *Rhincodon typus,* are the world's largest ectotherm, inhabiting tropical, sub‐tropical and temperate oceans circumglobally (Rohner et al. 2021). Juveniles of the species measuring 3–6 m are well studied due to the predictable aggregations that form on an often‐seasonal basis at approximately 50 sites around the world (Araujo et al. 2022; Womersley et al. 2024a). Many of these sites—termed ‘constellations’—are male biased, meaning that adults and females of the species are less well studied. Even less is known about the smallest individuals: whale shark neonates that measure < 1.5 m in length. The rarity of neonates, adult females and particularly pregnant females has made understanding whale shark reproductive ecology challenging. Because of a declining population and endangered status according to the International Union for Conservation of Nature (IUCN) Red List (Pierce and Norman 2016), it is important to explore the spatial dynamics of whale shark reproduction so that these key demographics and their critical habitats can be protected. In addition, understanding how climate change may potentially increase the exposure of sharks to anthropogenic threats such as shipping in the future is an important topic for informing conservation initiatives (Womersley et al. 2023; Womersley et al. 2024b).

Current evidence points to female whale sharks reaching maturity at 9–10 m total body length and first reproducing at ~30–40 years, whereupon they produce a litter of 250–350 embryos of an equal sex ratio which hatch inside the mother's twin uteri and feed on a yolk‐sac until being born free swimming (Pierce et al. 2021). It is still not known how frequently pregnancy occurs in an individual female, but the reproductive interval may be biennial or longer, with potential for rest periods as with other sharks (Pierce et al. 2021). There is likely substantial maternal energy input to developing embryos such that they are born free‐swimming at ~5% of maternal length, whereupon they are solitary and must fend for themselves. Neonate whale sharks are encountered by humans extremely rarely until they reach ~3 m when the males begin to frequent well‐known coastal constellation sites. The primary focus during these early stages is likely to be rapid growth necessitating high rates of energy intake while avoiding predation (Pierce et al. 2021; Rohner et al. 2021). Where parturition occurs, which habitats neonates use in their first months or years, and what they feed on at this time, remain open questions. Given that neonates receive no postnatal investment, selection of optimal habitat for their young is presumably as far as breeding females are able to extend their care. Under this scenario, areas with high densities of individuals under the age of 1 year may exist, where multiple females select similar environments to maximise the odds of neonatal survival by providing protection from predators and access to food (Heupel, Carlson and Simpfendorfer 2007). These ‘nursery’‐like areas may extend across vast areas of open ocean which makes speculation about their spatial dynamics difficult.

In a first step to address these unknowns, here we collate neonate whale shark sightings from the literature and map their geographical locations in relation to oceanography. With the discovery of potential oceanographic similarities in occurrence between oceans we discuss two potential pupping strategies: (i) an adaptive approach where females select dynamic oceanographic features for birthing young in batches that provide refuge from predators and enhance foraging opportunities, and (ii) a random approach where females birth arbitrarily in the open ocean and young are widely distributed, but that certain oceanographic features increase the likelihood of their capture by fishers or observation by other ocean users.

## 2 Methods

Literature searches yielded 33 whale shark neonate occurrence records from the last ~50 years where the geographic location was clearly noted or estimated (Table 1). These records were collected from peer‐reviewed literature and news reports, mostly from opportunistic capture of neonates where live, free‐swimming individuals were landed and later released. The geographic locations were mapped globally and then overlaid on maps of key environmental variables including sea surface temperature (SST, °C), Chlorophyll‐a (Chl‐a, mg m^3^), east/westward sea water velocity (converted into a single unit of geostrophic sea water velocity [UV, m^s−1^]), and dissolved oxygen concentration (DO, μmol L^−1^), chosen due to their potential importance as drivers of whale‐shark space use (Womersley et al. 2024b).

**TABLE 1:** Confirmed records of neonate whale shark sightings which included a geographic location from peer‐reviewed literature and news reports.

Date	Size (m)	Sex	Location (Lon, Lat)	Encounter type	Reference
12/08/1970	0.55	F	131, 10	Bycatch	Wolfson 1983
04/06/1971	0.63	F	−89, 8	Bycatch	Wolfson 1983
29/01/1975	0.93	F	−110, 10	Bycatch	Wolfson 1983
01/01/1976	0.59	F	−20, 8	Free swimming	Kukuyev 1996
27/02/1976	0.62	M	−93, 12	Bycatch	Wolfson 1983
01/01/1978	0.56	M	−10, 0	—	Wolfson 1983
01/01/1980	0.56	M	−11, 0	—	Wolfson 1983
01/02/1980	0.57	M	−12, 0	Bycatch	Wolfson 1983
01/08/1989	0.62	—	58, 24	Free swimming	Martin 2007
01/01/1991	—	—	114, −22	Multiple free swimming	Martin 2007
01/01/1993	0.61	—	57, −19	Alive in stomach	Colman 2002
01/01/1998	1	—	75, 8	Multiple free swimming	Martin 2007
01/01/2000	0.59	—	65, 25	Bycatch	Rowat et al. 2007
01/01/2000	—	—	65, 25	Bycatch	Rowat et al. 2007
15/11/2001	0.94	M	76, 11	Bycatch	Manojkumar 2003
01/05/2005	1.4	—	121, 13	Free swimming	Aca and Schmidt 2011
16/03/2006	1.13	—	92, 20	Bycatch	Rowat et al. 2007
01/12/2007	1.19	—	78, 9	Landed	Akhilesh et al. 2013
17/05/2008	1.15	—	76, 10	Landed	Akhilesh et al. 2013
07/03/2009	0.46	—	124, 13	Landed	Aca and Schmidt 2011
27/03/2009	0.64	M	124, 13	Bycatch	Aca and Schmidt 2011
01/11/2010	1.48	—	76, 10	Landed	Akhilesh et al. 2013
01/04/2011	0.95	—	76, 10	Landed	Akhilesh et al. 2013
05/03/2013	0.6	—	70, 21	Bycatch	Premjothi et al. 2016
15/03/2013	0.6	—	70, 21	Bycatch	Premjothi et al. 2016
06/04/2013	1	—	71, 21	Beached	Premjothi et al. 2016
27/10/2013	0.78	M	122, 13	Free swimming	Hsu, Lin and Joung 2014
29/04/2015	0.8	F	−81, −7	Bycatch	Pajuelo et al. 2018
13/02/2017	0.61	M	−81, −8	Bycatch	Pajuelo et al. 2018
11/03/2017	0.6	—	−81, −8	Bycatch	Pajuelo et al. 2018
15/03/2020	0.6	M	124, 13	Free swimming	Miranda et al. 2021
—	0.56	M	−30, 3	Alive in stomach	Kukuyev 1996
—	—	—	70, 20	Free swimming	Premjothi et al. 2016

For each oceanographic variable, a randomisation test was used to examine whether observed neonate locations occurred more often in specific environmental conditions compared with other potential locations where they could have been sighted. For every neonate occurrence, 100 randomised positions were generated within the IUCN range of the species and approximately 370 km from the coast (i.e., within Exclusive Economic Zones waters given that most individuals were found within these areas, 79%, *n* = 33). Then, SST and UV were extracted from the Copernicus Marine Service Global Ocean Physics Reanalysis (GLOBAL_MULTIYEAR_BGC_001_030) product for each observed and randomised location. This was also done for Chl‐a at 0 m and 100 m depth, and DO at 0 m and 100 m depth from the Copernicus Marine Service Global Ocean Biogeochemistry Hindcast (GLOBAL_MULTIYEAR_BGC_001_029) product. SST, Chl‐a, UV and DO extracted from the observed locations were compared to an equal number of randomised locations using a Wilcoxon rank‐sum test for 100 iterations. A relationship was considered a significant when > 75% runs had a *p* value < 0.05 (Queiroz et al. 2019).

A Generalised Additive Model (GAM) was used to explore the relationship between DO at 100 m and Chl‐a at 0 m depth based on the results of the Wilcoxon rank‐sum tests. A binomial GAM was run with a restricted maximum likelihood method including the fixed effects of logged Chl‐a and DO at 100 m and their interaction using the ‘mgcv’ package (Wood 2001) in R (R Core Team 2022). Here observed neonate occurrences (1) were modelled against randomised locations (0) following a presence‐absence ratio of 1:10 (Womersley et al. 2024b). The model was then used to identify regions globally with oceanographic conditions similar to those where neonates have been sighted to date, by using model parameters to predict probabilities of an occurrence based on averaged maps of DO and Chl‐a from 2005 to 2019.

## 3 Results

**FIGURE 1:**
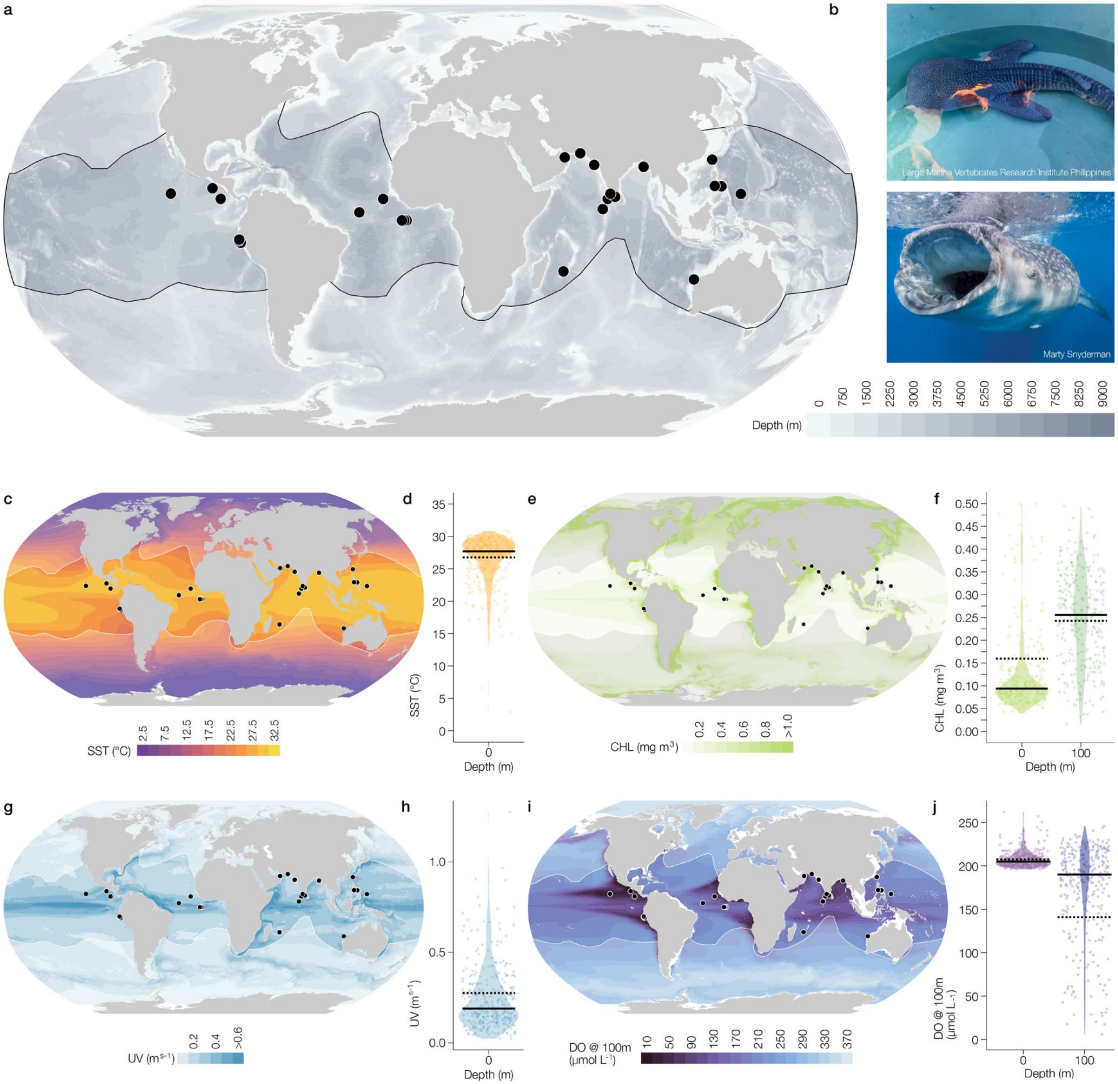
(a) Recorded sightings of whale shark neonates globally (black points). (b) Image of neonatal and adult whale sharks from Large Marine Vertebrates Research Institute Philippines group (b) and Marty Snyderman (b). (c) Whale shark neonates and sea surface temperature (SST, °C averaged for 2005–2019). (d) SST recorded from neonate locations and 10 randomised runs. (e) Whale shark neonates and Chlorophyll‐a (Chl‐a, mg m^3^ averaged for 2005–2019). (f) Chl‐a at 0 and 100 m depths recorded from neonate locations and 10 randomised runs. (g) Whale shark neonates and current velocity (UV) at 0 m depth (ms^−1^, averaged for 2005–2019). (h) UV recorded from neonate locations and 10 randomised runs. (i) Whale shark neonates and dissolved oxygen (DO) concentration at 100 m depth (μmol L^−1^, averaged for 2005–2019). (h) DO concentrations at 0 and 100 m depths recorded from neonate locations and 10 randomised runs. In (d, f, h) and (j) each point represents a single sighting or randomised location, the solid black line is the median value of randomised locations, the dashed black line the median of the observed sightings for each depth, and the violin displays the density distribution of all locations. IUCN whale shark distributions are shown in white in each map and with a black outline in (a).

Mapping the reported positions of 33 neonate whale sharks showed that locations were distributed in the west and east Pacific Ocean, the east Atlantic, and in the northern Indian Ocean (Figure 1a) in surface waters warmer than 22°C (Figure 1c). Most sightings of neonates in the Pacific and Atlantic oceans were in eastern equatorial regions close to productive eastern boundary currents (Figure 1e) and known permanent oxygen minimum zones (OMZs) (Figure 1i). The pattern of association with an OMZ was also evident in the Indian Ocean, where the region of low DO occurs in the north rather than the east. Although very low DO characterising an OMZ was not present in the western Pacific, neonate locations were in close proximity to DO concentrations < 170 μmol L^−1^ (or 3.83 mL L^−1^, Figure 1i).

Randomisation tests comparing environmental conditions in recorded neonate locations with feasible random locations yielded significant differences of < 0.05 on 7% of runs for SST (Figure 1d), on 95% and 1% of runs for Chl‐a at the surface and 100 m depth, respectively (Figure 1f), and on 23% of runs for UV at 0 m (Figure 1h). For DO at 0 m depth significant differences of < 0.05 were reported on 50% of runs, and on 86% of runs at 100 m depth (Figure 1j). The concentrations of Chl‐a at the surface and DO at 100 m depth are therefore significantly higher and lower, respectively, where neonates occurred compared to random locations within their known range. Averaged from 2002 to 2022, results showed that 9.4% of the global ocean contained DO concentrations at 100 m depth < 150 μmol L^−1^ (3.34 mL L^−1^, at the 0.25 resolution scale), while 55% of neonate sightings occurred in these waters.

**FIGURE 2:**
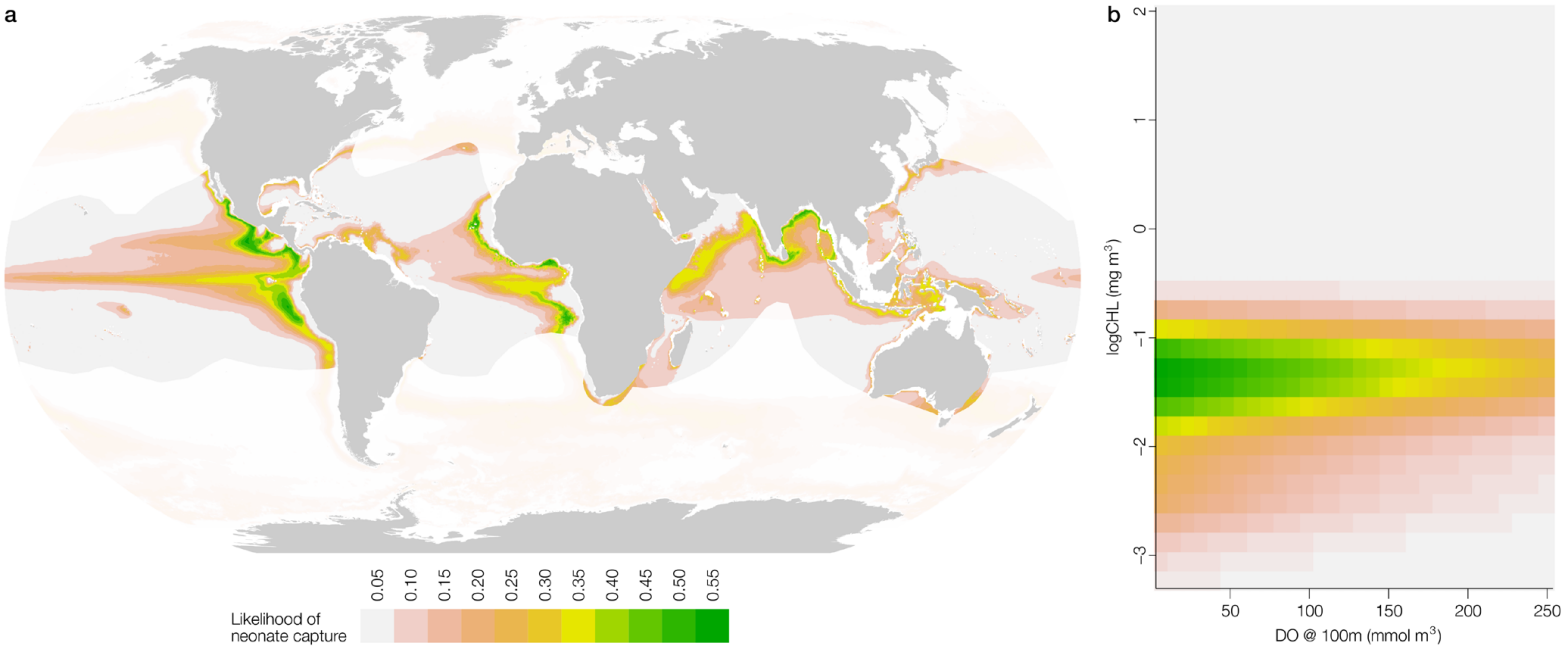
(a) Locations of environments that have similarities to those where whale shark neonates have been encountered. Green represents DO‐at‐depth (100 m) and surface Chl‐a values where the likelihood of a neonate being sighted is higher than pink and white areas based on a Generalised Additive Model. (b) The interaction between DO‐at‐depth (100 m) and surface Chl‐a displaying higher (green) and lower (white and pink) likelihood of a whale shark neonate being sighted in environmental space.

As a first step to identify habitats that have similarities to those where whale shark neonates have been encountered to date, a GAM was used to explore the influence of Chl‐a, DO at 100 m and their interaction, on neonate sighting location. In environmental space, the model predicted neonates to be encountered most likely in mid‐range Chl‐a at the surface and lower DO‐at‐depth (Figure 2b). In geographic space, the model predicted the highest encounter likelihood of neonates in waters above OMZs in all major ocean basins, focussed around intermediate Chl‐a regions at the boundaries of high Chl‐a hotspots associated with eastern boundary upwellings in the Atlantic and Pacific and Monsoon driven upwellings in the Indian Ocean (Figure 2a). For example, offshore waters in the Bay of Bengal and Arabian Sea in the Indian Ocean, Angola, the Gulf of Guinea and Cape Verde in the Atlantic, and Peru, Guatemala and Philippines in the Pacific—among others—were the most likely areas to encounter neonates based on the GAM given their similarities to areas where neonates have been sighted to date (Figure 2a).

## 4 Discussion

Global surface observations of neonate whale sharks appear to be associated with intermediate Chl‐a at the surface and lower DO concentrations at depth more often than expected compared to random chance. In addition, the distribution of major OMZs co‐occurred with the majority of neonate sightings which were not associated with SST or UV. It is possible that observed neonate locations reflect aggregation sites of whale sharks within their range. However, known aggregation sites globally (Womersley et al. 2024a) do not directly co‐occur with neonate sightings and indeed are not limited to areas above OMZs or lower DO waters. Here, two general hypotheses are proposed that could explain this apparent environmental association. Firstly, mid‐range Chl‐a areas located above OMZs could be open ocean ‘nurseries’ providing enhanced foraging opportunities for newborn whale sharks whilst acting as an oceanic refuge away from the most productive areas with higher predator density. Second, OMZs and other areas with low DO at depth could vertically compress the habitat of neonates leading to more likely near‐surface capture or observation by humans. Both explanations potentially lead to a greater probability of surface observations of these young sharks within these specific areas.

Under the first proposed hypothesis mature females may employ an adaptive pupping strategy, selecting OMZs for giving birth in discrete batches that provide refuge from predators and foraging opportunities, and thus limiting potential mortality risk both spatially and temporally. Permanent OMZs—comprising ~8% of the ocean surface (~30 million km^2^) with a core volume of ~10 million km^3^ (Paulmier and Ruiz‐Pino 2009)—primarily occur along the eastern boundary regions between oceans and continental landmasses associated with coastal upwelling (such as in the eastern tropical Atlantic and Pacific Oceans and in the northern Indian Ocean, although here upwelling is linked to Monsoon winds as opposed to eastern boundary regions [Chan 2019, Vinayachandran et al. 2021], Figure 1g). They are highly productive areas characterised by severe hypoxia (< 22.2 μmol L^−1^, 0.5 mL L^−1^) (Diaz and Rosenberg 2008). With increasing depths, DO within core OMZ waters initially decreases within the mesopelagic before increasing, with the lowest oxygen waters usually found between 200 and 1000 m deep, although this varies between regions (Paulmier and Ruiz‐Pino 2009; Gallo and Levin 2016). DO can have profound effects on biodiversity (Breitburg et al. 2018), such as the zooplankton communities on which whale sharks directly feed (Rohner and Prebble 2021). At the lower boundary of OMZs, steep oxygen gradients create microhabitats of differing DO that are characterised by layers of high zooplankton biomass and abundance (Saltzman and Wishner 1997; Wishner et al. 2013) with much of the midwater zooplankton biomass and activity in OMZ regions focused within oxycline boundary communities (Steinberg et al. 2008). In the Arabian Sea, for example, aggregating zooplankton at the lower OMZ oxycline results in higher levels of biomass than at similar depths where the OMZ is weaker or absent (Madhupratap and Haridas 1990; Vinogradov and Voronina 1962; Wishner, Gowing and Gelfman 1998). At their upper boundary, higher zooplankton biomass also aggregates within a narrow band, associated with more oxygenated surface waters (Tutasi and Escribano 2020).

These OMZ boundary areas could provide optimal conditions for whale shark neonates, and may, at broad scales, come close to meeting the criteria for a pelagic ‘nursery’‐like habitat (Heupel, Carlson and Simpfendorfer 2007). In addition, for whale shark young transiting the prey sparse or predator‐rich open ocean for ~3 years, the zooplankton aggregating nature of the upper and lower OMZ boundaries may provide optimal conditions for foraging in productive waters whilst allowing individuals to remain in close proximity to ‘hypoxic shelter’ in the form of physiological refuge from predators. Hypoxic waters within OMZs could provide ideal protection for neonates given they may exert aerobic constraints on relatively high oxygen demand predators such as blue shark, *Prionace glauca,* and blue marlin, *Makaira nigricans* , both of which have been known to occupy waters above OMZs and prey on whale shark young (in the Atlantic and Indian Oceans [Rowat and Brooks 2012]). Furthermore, whale shark neonates have been found alive in stomachs of both these predator species suggesting neonates may be capable of surviving in hypoxic conditions at least for short periods. Their disruptive camouflage suggests that these small sharks occupy waters near the surface (most likely to regulate their body temperature given their underdeveloped capacity for thermal inertia; Nakamura, Matsumoto and Sato 2020) for at least some of their time, where they must employ strategies to avoid visual predators. Similar habitat selection strategies are known for other shark species. For example, juvenile sandbar sharks, *Carcharhinus plumbeus* , select low DO environments, linked to both predator avoidance and prey encounter rates, where occupying waters just outside the hypoxic zone provides juveniles with the opportunity to make short forays inside hypoxic areas to forage on fish that are more hypoxia tolerant than they are (Crear et al. 2020). The observed preference for moderate Chl‐a areas also suggests a strategy to maximise foraging and minimise predation compared to high Chl‐a areas, where predators will be most abundant. Acting together, these features maximise the odds of neonatal survival by providing protection from predators and access to food (Heupel, Carlson and Simpfendorfer 2007).

An alternative hypothesis potentially explaining the observed association between early whale shark life stages and the low DO found in OMZs, is that mature females give birth arbitrarily in the open ocean and young are widely distributed, but that OMZs increase the likelihood of their capture by fishers or observation by other ocean users. Under this proposed scenario, neonates occupying waters around OMZs may be displaced into shallower surface waters by relative intolerance of DO levels at depth, resulting in them being more likely to be encountered or captured by humans. This is perhaps a more established hypothesis and relates to the widely discussed phenomenon of vertical habitat compression, where marine life is shoaled into a narrower (than normoxic areas) vertical band of water above low DO areas (Gruber et al. 2021; Laffoley and Baxter 2019). This vertical habitat compression has been demonstrated clearly in blue sharks in the eastern tropical Atlantic OMZ, where factors associated with low DO contributed to vertical habitat compression and increased susceptibility to capture by surface fisheries (Vedor et al. 2021). In this case, greater intensity of longline fishing effort occurred above OMZ waters compared to adjacent areas and higher shark catches were associated with strong DO gradients, suggesting potential aggregation along DO boundaries contributed to habitat compression and higher fisheries capture in this species (Vedor et al. 2021).

A possible aerial report of a whale shark giving birth near Ningaloo Reef, Western Australia (Martin 2007), supports the hypothesis that young are birthed randomly as this site was not located near a permanent OMZ. Here, 14 apparently new‐born whale sharks were seen emerging at the surface alongside a large female (Martin 2007). This appears to be one of the only records of a whale shark supposedly giving birth and circumstances such as premature abortion or incorrect identification cannot be ruled out (e.g. cobias, *Rachycentron canadum* , which can be mistaken for neonates due to their size and appearance are known to swim alongside whale sharks [Akhilesh et al. 2013]). Pupping in such a location where adults of the species are often feeding on prey that may be too large for the neonates to consume—for instance chaetognaths, euphausiids, copepods, stomatopod larvae and small fishes, with the euphausiid, *Pseudeuphausia latifrons,* forming the most significant component of their diet within the region with a size range 8–13 mm—raises further questions. As does pupping in potentially predator‐rich surface waters with seemingly limited areas for refuge. In addition, strandings of juvenile, sub‐adult and adult whale sharks in proximity to low DO areas, such as along the Andhra Pradesh coastline, India could be linked to a temporally dynamic shallowing OMZ in the Bay of Bengal, potentially forcing these individuals into sub‐optimal environments for prolonged periods (Gallo and Levin 2016; Masood 2022). This would suggest that they are not able to inhabit or make use of such extreme habitats for reproductive purposes.

Other features may be involved in whale shark birthing habitat selection. For example, bathymetric features like remote islands, atolls, seamounts and pinnacles may play an important role in whale shark reproduction in that they offer a fixed refuge for young and orientational or navigational waypoints for adult females. The high number of adult females in areas like the Galapagos, Ecuador in the Pacific (Hearn et al. 2016) and Fuvahmulah, Maldives in the Indian Ocean (Chloe Winn, personal communication) supports this hypothesis. Although we explored current velocity at broad scales and found no correlation with neonate occurrence, it is entirely possible that neonates do get caught in local currents which influence their movements due to their poor swimming abilities. It is also possible that neonates get ‘caught’ in upwelling systems forcing them into surface waters, although this phenomenon would not explain many of the locations in the Indian Ocean as here the strongest, Monsoon driven upwelling occurs on the Somali coast (Vinayachandran et al. 2021). However, upwelling also occurs on the Oman coast and southwest coast of India where neonates have been recorded (Vinayachandran et al. 2021), and the lack of records in Somalia could instead be linked to reduced numbers of ocean users and reporting networks in the region. If currents and upwelling systems do influence the distributions of whale shark young, it is difficult to speculate about the areas where the adult females are selecting or indeed if there is any selection involved in the birthing process. As such, the question as to whether whale sharks select for specific environments still remains open and future studies are needed to more formally address the hypotheses suggested here, and others too potentially.

### 4.1 Future Directions

Whale shark reproductive ecology requires considerable further exploration. As a first step, a simple model of neonate occurrence was developed here to help identify potential habitats and to focus observation efforts in future. The model indicates that there is a higher likelihood of neonate occurrence in ocean areas with both intermediate surface Chl‐a and low subsurface DO concentrations. Whilst the number of environmental variables used in the model was restricted to the two most significantly associated with neonate presence according to our randomisation tests, it serves as a first order approximation of the ocean areas and habitat types most likely to support observations of neonates, potentially directing future studies. For example, within these regions, use of cutting‐edge technology can help gather the data needed to test fully the two hypotheses in the wild, or indeed develop new hypotheses. Recent advancements in DO sensing tags—capable of measuring real‐time dissolved oxygen levels experienced by marine animals—hold immense possibility (e.g., da Costa et al. 2024). By deploying oxygen sensing tags onto whale sharks combined with recorded tri‐axial accelerometry for fine‐scale body movements, unprecedented insights into their behavioural responses to DO concentrations are possible (da Costa et al. 2024). In particular, biologging within OMZs could be used to establish whether adult females can move throughout low DO environments and whether they choose to occupy these areas for extended periods. Similarly, biologging devices small enough to deploy onto neonatal whale sharks—like those deployed on juvenile sea turtles (Candela et al. 2024)—would also improve our understanding of their space use and physiological capabilities. These require innovation and given the limited sightings of these sharks it seems unlikely that this demographic stage can be regularly targeted for such tagging. Although the whale shark neonates were sighted by ocean users in surface waters or captured at an unknown depth, it was assumed here that they can access depths of at least 100 m. Adults and juveniles of the species can dive to > 1000 m, (Brunnschweiler et al. 2009), but whether small neonates are able to make forays into deeper waters is unknown and could be addressed with such technology. More generally, deep, subsurface oceanographic conditions can influence shallower, surface layer ecology through processes such as prey compression (Waller et al. 2024) and can therefore drive surface dwelling animal movements and behaviours indirectly.

Broader tracking or genetic studies across ocean basins will help to identify whether whale sharks from different constellations or regions do traverse long distances to use the same pelagic areas for pupping (Schmidt 2021). Such a result would be important for conservation, as it suggests that those whale shark constellations already depleted by overfishing, shipping and other human pressures can still be repopulated from other areas over time (Rohner et al. 2021). Increased broad‐scale tracking may also help to establish whether individual females have the capacity for philopatry, returning to similar sites to give birth in subsequent years. In addition, social media is an important tool for sharing sightings or discrete events involving wildlife, and while no social media accounts were included here due to lack of location information, the tool could be searched more thoroughly in future and used to compile a wider database of neonate sightings, for whale sharks and other shark species (Cranswick et al. 2022; Mannocci et al. 2022).

Improving our understanding of whale shark behavioural responses to DO has important conservation implications as expanding OMZs in future may increase the likelihood of negative interactions with human activities, such as shipping and fisheries. If this species spends more time in oxygenated surface waters while transiting over OMZs this may increase the risk of ship collision or fisheries by‐catch (Waller et al. 2024; Womersley et al. 2024b) especially in areas where low DO at depth and intense human activities co‐occur, for example in the eastern tropical Atlantic and Pacific. Knowledge of areas that are key to early life stages will be also important for informing global conservation planning initiatives such as resolutions to re‐route shipping away from whale shark constellations and potentially birthing areas (Womersley et al. 2023), or for the designation of Important Shark and Ray Areas (Hyde et al. 2022).

## Author Contributions

**Freya C. Womersley:** conceptualization (equal), data curation (lead), formal analysis (lead), investigation (lead), methodology (lead), project administration (lead), visualization (lead), writing – original draft (lead), writing – review and editing (lead). **Matt J. Waller:** conceptualization (supporting), formal analysis (supporting), writing – review and editing (supporting). **David W. Sims:** conceptualization (equal), funding acquisition (lead), supervision (lead), writing – review and editing (supporting).

## Conflicts of Interest

The authors declare no conflicts of interest.

## Data Availability

All data used in this study is freely available from online datasets. Environmental data was sourced from Copernicus Marine Service (https://marine.copernicus.eu/), and neonate sightings were sourced from published literature included in Table 1.
